# Insights into Recent Updates on Factors and Technologies That Modulate the Glycemic Index of Rice and Its Products

**DOI:** 10.3390/foods12193659

**Published:** 2023-10-04

**Authors:** Tai Van Ngo, Kannika Kunyanee, Naphatrapi Luangsakul

**Affiliations:** School of Food Industry, King Mongkut’s Institute of Technology Ladkrabang, Bangkok 10520, Thailand; 64608035@kmitl.ac.th (T.V.N.);

**Keywords:** rice, glycemic index, advanced technologies, product, digestibility

## Abstract

Rice is a staple food and energy source for half the world’s population. Due to its quick digestion and absorption in the gastrointestinal tract, rice is typically regarded as having a high or medium–high glycemic index (GI); however, this can vary depending on the variety, nutrient compositions, processing, and accompanying factors. This report included a table of the glycemic index for rice and rice products in different countries, which could give an overview and fundamental information on the recent GI of different rice varieties. In addition, latest updates about the mechanism effects of rice nutritional profiles and processing techniques on GI were also provided and discussed. The influence of state-of-the-art GI regulation methods was also evaluated. Furthermore, the effectiveness and efficiency of applied technologies were also given. Furthermore, this review offered some aspects about the potential nutraceutical application of rice that food scientists, producers, or consumers might consider. Diverse types of rice are grown under various conditions that could affect the GI of the product. The instinct nutrients in rice could show different effects on the digestion rate of its product. It also revealed that the rice product’s digestibility is process-dependent. The postprandial glucose response of the rice products could be changed by modifying processing techniques, which might produce the new less-digestive compound or the inhibition factor in the starch hydrolysis process. Because of the significant importance of rice, this paper also concluded the challenges, as well as some important aspects for future research.

## 1. Introduction

According to the World Health Organization, at least 347 million people are suffering from diabetes. And this number is increasing day by day; it is predicted that by 2030, this number could reach double the current number [1]. Diabetes mellitus, more commonly referred to as diabetes, is a group of metabolic diseases that are characterized by the patient’s increased blood sugar level [2]. In recent years, research has shown the positive link between diabetes and food consumption, especially starch-based food. Due to the reduced glucose metabolism during the postprandial phase, it is advised for diabetics to consume low glycemic index (GI < 55) meals. As a result, GI is seen as crucial factors in selecting diets for diabetic people [3]. The postprandial blood glucose response to diets high in starch and sugar has been explained using the GI concept [4]. The glycemic load (GL) is a measure that combines the glycemic index (GI) of a specific item with its accessible carbohydrate content, providing an assessment of both the average quality and quantity of carbohydrates present in the diet [3]. Consuming a diet that is high in glycemic index (GI) has the potential to induce a swift elevation in blood glucose levels. Furthermore, prolonged adherence to a high-GI or glycemic load (GL) diet may lead to the development of hyperglycemia, glucose intolerance, and hyperinsulinemia. There is evidence suggesting a potential association between a diet with a high glycemic index/glycemic load (GI/GL) and an increased risk of many chronic diseases, including coronary heart disease, diabetes, obesity, and some types of cancers [5,6]. To understand digestive characteristics, various methods have been developed both in vitro and *in vivo*. However, depending on the type of product, the analytical method is also different or adjusted to suit actual conditions.

A staple food for almost 4 billion people worldwide, rice supplies 15% of protein and 21% of energy per person globally [7]. In South Asia, Southeast Asia, Africa, and Latin America, rice is farmed in a variety of agro-ecosystems. In addition, one of the most significant staple foods in the world is rice (*Oryza sativa* L.), which contains 60–80% starch depending on the variety [8]. It is a vital staple food and a good source of macronutrients, contributing to 14% of protein and 2% of fat [9,10]. However, rice is considered to have a medium to high glycemic index in general [7]. The nutritional content of the grains varies among all rice varieties due to differences in appearance at the macro and micro levels, which also lead to differences in the glycemic index value. In addition, the geographical location and the presence of antioxidant components are also one of factors that strongly influence the GI value of rice and its processed products. Such factors are also less clearly mentioned in the GI tables of rice and rice products in the study by Kaur et al. [7]. However, not only intrinsic factors, such as rice starch composition, starch structure, macronutrients content, and the presence of polyphenols and fiber, affected the GI of the rice products [11,12], but the production and processing of rice products also significantly contributes to the difference in digestion behavior, known as the extrinsic factor. The digestion behavior depends on various factors, including the cooking methods, preparation processing, and the presence of other food components [13]. However, the mechanism of each factor affecting the glycemic index of rice and rice products also differs. This indicates the need to understand the impact of internal and external factors on the digestion of rice and rice products. From these perspectives, advanced and novel methods could be developed to modulate the digestibility of rice and rice products.

In recent years, many methods of reducing the GI of rice have been extensively studied, which can improve on previously developed methods or newly developed methods. Parboiling, germination, and polishing with various conditions were commonly applied to modulate the GI of rice grain [14,15,16,17]. Meanwhile, the gamma irradiation process was also recently reported, which could reduce the digestion rate of rice [18]. Physical modification is now considered a trend in the modification method. Some of the methods, such as heat-moisture treatment, ultrasound, microwave, and high-pressure homogenization, were operated to lower the GI of rice flour and rice starch [19,20,21]. In addition, the complexation with different phytochemical sources or fortification of the low-GI ingredients was also examined to reduce the GI of rice products [22]. However, the effectiveness of each method in reducing digestion rate could differ due to the product types and nutritional values. The objective of this review is therefore to present the recent updated information about the GI table of different rice and its product, as well as the recent measurement method. Furthermore, the mechanism and processing impact on the glycemic index of rice are also reviewed to give further recommendations for this abundant and valuable source. This review summarizes the information in the English articles that have already been recently published about rice and its product related to the glycemic index on the Web of Science and Scopus databases up to December of 2022 and categorizes them into four main sections: (i) basic information about starch digestion and recent advanced measurement method; (ii) the mechanism effect of various factors on GI; and (iii) recent advanced technologies to modulate GI. All readers with academic backgrounds could obtain insight into the multifaceted issues surrounding rice composition, digestion, mechanisms, and the nutraceutical perspective by the comprehensive approach of glycemic reduction in rice products presented in this review.

## 2. Starch Digestion and Glycemic Index

### 2.1. Starch Digestion Process

As a natural form of carbohydrates, starch is one of the main sources of energy in the human diet. Chemically, amylose and amylopectin are the two forms of polyglucans that form starch. Amylose typically takes the form of a linear polysaccharide made up of D-glucose units connected by α-(1-4)-glycosidic linkages. While amylopectin is considerably larger than amylose and extensively branched, it also has α-(1,6)-glycosidic branch points in addition to α-(1-4)-glycosidic linkages [11].

In the human body, disaccharides are first formed by the hydrolysis of starch by salivary and pancreatic amylase enzymes in the mouth and small intestine, respectively. The first product of this process is usually maltose and a small amount of dextrin, which is further broken down to glucose by the two enzymes maltase-glucoamylase and sucrase-isomaltase at the brush border [3]. The end product of hydrolysis is glucose (monosaccharides), which is further absorbed into the bloodstream. Therefore, the degree of starch digestibility is closely related to the amount of glucose absorbed in the blood. In addition, insulin, a hormone produced by the β-cells of the pancreas, plays a significant role in promoting sugar consumption and maintaining the level of blood glucose. Excessive release of insulin depletes β-cells, which is the main cause of diabetes [23]. Meanwhile, glucose is one of the vital precursors that promote the release of insulin. The control of releasing glucose during digestion is preferable for diabetics to have a slow release of reducing sugars. However, real food systems contain not only starch, but also other nutrients. In addition to the enzyme lipase from the pancreas, pepsin in the stomach also contributes to the digestion process to digest lipids and protein, which also further affects the difference in the glycemic index of food [24].

### 2.2. Glycemic Index and Current State-of-the-Art Estimation Method

The glycemic index (GI) is a method used to classify dietary carbohydrates based on their impact on the blood glucose response after intake of starch- or sugar-rich meals. It was first introduced by Jenkins et al. as follows: “*The GI is defined as the incremental blood glucose area following the test food, expressed as the percentage of the corresponding area following a carbohydrate equivalent load of a reference product*” [4]. According to this idea, a diet with a lower GI may result in slower rates of digestion and absorption, which may decrease the rapid rise in postprandial hyperglycemia and insulin concentration, as well as subsequently have an impact on the treatment of diabetes [3]. Moreover, Englyst et al. [2] previously investigated the relationship between GI and starch digestion rate. Rapidly digestible starch (RDS), slowly digestible starch (SDS), and resistant starch (RS), which is hydrolyzed within the first 20, hydrolyzed between 20 and 120 min, and remains undigested after 120 min, respectively, were divided into fractions that differ in their rate of hydrolysis in the small intestine.

Different in vitro protocols were applied for the determination of the GI of rice and rice products, which mainly depended on the purpose and goal of the studies. The method of Goñi et al. [25] was widely applied to investigate the estimated glycemic index of food as an in vitro protocol. However, in some cases, due to the laboratory conditions and food matrix, the method was improved with/without slight modification from the Goñi method or the newly developed method. It could be seen that the common digestive enzymes used are pepsin, pancreatic α-amylase (AA), and amyloglucosidase (AMG). In general, the time for hydrolysis is typically about 180 min in 30 min intervals. The released glucose at each taken sample point was quantified as a percentage of hydrolyzed starch. Taken from a plot of these values is the area under the digestion curve (AUC) of the test food, whose ratio (expressed in percentage) with the AUC of the reference food is referred to as the hydrolysis index (HI). The equation commonly used for calculating predicted glycemic index of sample is “eGI = 39.71 + (0.549HI)”, especially rice and rice products [26,27,28,29].

Different digestion conditions, such as enzyme activity, incubation durations, and the change in pH, were considered in several alternate protocols as shown in Table 1. The INFOGEST approach is one of them; it is a static protocol created for a specific food, such as rice and their products [30], and was created to mimic the physiological *in vivo* parameters of the human gastrointestinal tract. It offers a quick and simple standardized method to analyze how food or pharmaceuticals behave during the digestion process. Utilizing this technology would result in less variance in the composition and structure of the food matrix than other methods, which would be advantageous [26]. Furthermore, the recent study by Srikaeo [31] reported a new and simple method to estimate the glycemic index of rice and rice flour with/without cooking that is suitable for farmers and food manufacturers with limited laboratory resources for field application.

The comparison of static and dynamic in vitro protocols for starch hydrolysis of rice pudding was also recently published by Li et al. [30], which indicated that only in the dynamic model showed the inhibitory impact of cinnamon on starch hydrolysis in the stomach and intestinal stages. For food to undergo structural and chemical changes, dynamic models must allow for progressive digestive secretions, pH evolution, and luminal transit. Dynamic models represent gut motility and the mechanical and hydrodynamic forces and conditions that occur from it, significantly influencing the physical and chemical environments of time during in vitro digestion. Nutrients’ bio-accessibility and bioavailability have been predicted using dynamic models, which has been useful [32]. Further study could apply this method to study the effect of micronutrients or other molecules on the starch hydrolysis rate of food.

The GI of rice product is also determined *in vivo*, which requires the use of animal or human subjects for evaluation of the two-hour blood glucose response after food intake. When investigated *in vivo*, the digestive process is better understood. However, this method has difficulties in terms of ethical considerations, intricacy, and tediousness [3]. Besides understanding the glycemic index, the glycemic load (GL) is also one of the important factors, which is a derived metric that is calculated by multiplying the GI of a product by the amount of carbohydrates in grams per serving, and then dividing the result by 100 [6]. A GL above 20 is regarded as high, whereas a GL ranging from 11 to 19 is considered medium, and a GL of 10 or below is classified as low. It is often observed that food items with a low GL in a standard serving size tend to have a correspondingly low GI [6]. The glycemic load is considered to be a more comprehensive and accurate indicator of the physiological effects resulting from the consumption of carbohydrates. The consideration of the glycemic index is incorporated, although the analysis encompasses a more comprehensive perspective beyond the only reliance on the index [33].

Regardless of time and money consumption, for animal testing, six-week-old Wister male rats, under formal testing ethical approval, were recently selected to measure the glycemic index of rice in Bangladesh [34]. At 0, 30, and 120 min after feeding, blood was taken from the tail vein and quantified using a digital glucose meter. After an overnight fast, the animals were given oral gavages of test rice suspension before the fasting glucose level was determined. Glucose solution was considered as the reference food.

The glycemic index was also measured in the human body. The early recommended method of the FAO/WHO Joint Expert Consultation [35] was applied to estimate the effect of processing conditions on the glycemic index of rice [17]. In addition, the recent study of [36], which examined the *in vivo* glycemic response of cooked parboiled rice, took the blood and measured the blood glucose level from 25 heathy volunteers (BMI = 18.5–23 kg/m^2^) after consuming standard and rice sample (0–120 min) on a separate day. The proportion of food that they consumed contained 50 g available carbohydrate. Fasting is required for the subjects and Human Ethics should be approved before testing. Pre-diabetic subjects were also considered to choose the appropriate rice for health promotion [29].

Rice is the second most extensively consumed cereal globally, following wheat. Rice serves as the primary dietary staple for over two-thirds of the global population. In Asia, a population exceeding 2 billion individuals relies on rice as their primary source of nutrition, constituting 80% of their energy requirements. Rice is composed of approximately 80% carbs, 7–8% protein, 3% fat, and 3% fiber. However, the percentage of carbohydrate, which is mainly related to the value of GI, differs by variety and geography [37]. For example, the carbohydrate content of seven rice varieties in India was about 72% [38]. “Cẩm” rice in Vietnam comprises about 80% of carbohydrate content [8,10]. The KDML105 and CN1 rice varieties in Thailand contained 73.76% and 71.44% of carbohydrate, respectively. There are numerous types of rice grains in existence, and they all produce postprandial blood glucose responses that differ significantly from one another. The glycemic index and the nutritional compositions database from different native forms of rice, rice flour, cooked rice, and their products from various countries through in vitro and *in vivo* tests are summarized in Table 1. According to the glycemic index (GI) research on rice products conducted all over the world, values range from 64 to 93. Different compositions, varieties or planting areas led to a variation in the glycemic index of materials. It could be seen that besides the macronutrient and appearance amylose content, the presence of endogenous phenolic compounds could also lead to a change in the digestion behavior of rice products. High polyphenol content could have the potential to produce medium to low-GI rice, which could be seen in the study of [39]. Understanding the initial GI value of rice could provide the fundamental and basic information to improve or lower glycemic index by various techniques.

**Table 1:** Recent published GI and nutritional data of rice grain (milled and brown), rice flour, and products from rice flour/starch.

Country/Region	Type of Food	Amylose Content (%)	Protein (%)	Lipid (%)	TPC (mg%GAE)	GI	Analysis Method	Ref.
Thailand	‘KDML105’ white rice flour	16.86	-	-	-	85.71 ± 0.21	*In Vitro*	[40]
	‘CN1’ white rice flour	26.50	-	-	-	82.73 ± 1.57		
	Rice pancake (Jasmine rice)	-	5.75	11.78	-	60.8 ± 1.8	*In Vitro*	[41]
	Hommali rice flour	21.3 ± 0.63			0.06 ± 0.01	87.1 ± 2.8	*In Vitro*	[42]
	Riceberry rice flour	15.3 ± 0.10			2.52 ± 0.03	65.4 ± 2.6		
India	Gluten-free rice cookies	26.27 ± 0.42	9.10 ± 0.31	40.00 ± 0.25	-	44.60 ± 0.48	*In Vitro*	[28]
	Broken white rice flour (variety: Lalat)	28.31 ± 0.30	10.15 ± 0.33	1.25 ± 0.15	-	50.12 ± 0.53		
	Cooked brown rice (Kattuyanam)	30.03 ± 0.09	-	-	5.99 ± 0.11	47.19 ± 3.2	*In Vivo*	[39]
	Cooked red rice (Red kavuni)	27.28 ± 0.26	-	-	5.89 ± 0.19	61.69 ± 4.0		
	Cooked black rice (Black kavuni)	27.30 ± 0.08	-	-	3.33 ± 0.02	56.27 ± 4.3		
	Cooked white rice (Karudan samba)	27.71 ± 0.04	-	-	1.91 ± 0.03	69.74 ± 4.5		
Philippine	Cooked milled IMS 2 rice	1.7 ± 0.2	6.8 ± 0.6	5.7 ± 0.2	-	95.8 ± 2.0	*In Vitro*	[43]
	Cooked milled NSIC Rc160 rice	13.3 ± 0.2	8.7 ± 0.7	4.7 ± 0.8	-	86.6 ± 1.6		
	Cooked milled IR64 rice	17.6 ± 0.1	8.9 ± 0.3	1.2 ± 0.1	-	76.6 ± 3.1		
	Cooked milled PSB Rc10 rice	24.0 ± 0.5	7.4 ± 0.2	1.8 ± 0.6	-	72.3 ± 2.3		
	Cooked brown IMS2 rice	-	6.3 ± 0.2	1.7 ± 0.4	-	76.4 ± 1.2		
	Cooked brown NSIC Rc160 rice	-	8.5 ± 0.3	0.9 ± 0.5	-	73.2 ± 1.3		
	Cooked brown IR64 rice	-	8.7 ± 0.3	0.9 ± 0.0	-	70.6 ± 0.6		
	Cooked brown PSB Rc10 rice	-	7.2 ± 0.3	1.6 ± 0.4	-	66.7 ± 1.1		
	Cooked milled IMS 2 rice	0.3	5.0	0.8	-	63 ± 2	*In Vivo*	[44]
	Cooked milled Sinandomeng rice	5.6	3.6	0.5	-	75 ± 4		
	Cooked milled NSIC Rc160 rice	6.5	3.6	0.4	-	70 ± 4		
	Cooked milled PSB Rc18 rice	7.6	3.1	0.6	-	59 ± 4		
	Cooked milled IR64 rice	9.4	3.5	0.4	-	57 ± 3		
	Cooked milled PSB Rc12 rice	8.0	3.2	0.4	-	63 ± 3		
	Cooked milled PSB Rc10 rice	10.1	3.1	0.4	-	50 ± 3		
	Cooked brown Sinandomeng rice	5.1	3.5	1.4	-	55 ± 2		
	Cooked brown IR64 rice	9.3	3.7	1.5	-	51 ± 1		
Bangladesh	Rice BRRI-Dhan-29	-	-	-	-	75.02	*In Vivo*	[34]
Sri Lanka	Cooked colored rice Cv. *Madathawalu*	24.83 ± 0.69	-	-	-	76.2 ± 0.9	*In Vitro*	[45]
	Cooked colored rice Cv. At 362	28.67 ± 0.82	-	-	-	72.6 ± 1.9		
	Cooked colored rice Cv. Sudu Heenati	28.42 ± 0.50	-	-	-	72.3 ± 0.9		
	Cooked colored rice Cv. Bw 272–6B	28.38 ± 0.67	-	-	-	71.9 ± 1.5		
	Cooked white rice Cv. Suwandel	24.96 ± 0.15	-	-	-	79.0 ± 0.8		
	Cooked white rice Cv. Bg 352	29.23 ± 0.72	-	-	-	78.8 ± 0.6		
	Cooked white rice Cv. Bw 267–3	30.19 ± 0.38	-	-	-	78.6 ± 1.3		
	Cooked white rice Cv. Bg 360	27.26 ± 0.2	-	-	-	77.6 ± 0.6		
Taiwan	Cooked white rice cv. TRGC9152	12.77 ± 0.24	3.75 ± 0.02	0.12 ± <0.01	-	60.18 ± 0.71	*In Vitro*	[46]
						73.1 ± 5.7	*In Vivo*	
	Cooked white rice cv. IR50	2.19 ± 0.04	2.88 ± 0.03	0.17 ± <0.01	-	63.55 ± 0.33	*In Vitro*	
						77.3 ± 4.1	*In Vivo*	
	Cooked white rice cv. Taichung Sen 17	11.60 ± 0.18	3.24 ± 0.05	0.10 ± <0.01	-	62.14 ± 0.82	*In Vitro*	
						77.3 ± 4.1	*In Vivo*	
	Cooked white rice cv. Taikeng 9	4.25 ± 0.01	2.94 ± 0.05	0.08 ± <0.01	-	78.60 ± 0.79	*In Vitro*	
						87.5 ± 4.3	*In Vivo*	
	Cooked white rice cv. Taichung Sen 10	3.90 ± 0.07	3.00 ± 0.05	0.09 ± <0.01	-	78.41 ± 0.41	*In Vitro*	
						82.5 ± 5.5	*In Vivo*	
	Cooked white rice cv. Khazar	2.08 ± 0.02	3.45 ± 0.04	0.13 ± 0.01	-	82.55 ± 0.19	*In Vitro*	
						88.9 ± 4.1	*In Vivo*	
	Cooked brown rice cv. Taikeng 9	1.78 ± 0.09	4.96 ± 0.02	1.89 ± 0.02	-	58.01 ± 0.85	*In Vitro*	
						70.8 ± 4.3	*In Vivo*	
	Cooked brown rice cv. Taichung Sen 10	3.90 ± 0.13	4.82 ± 0.06	1.74 ± 0.02	-	58.01 ± 0.85	*In Vitro*	

## 3. Factor Affecting the Glycemic Index of Rice and Their Products

**Figure 1:**
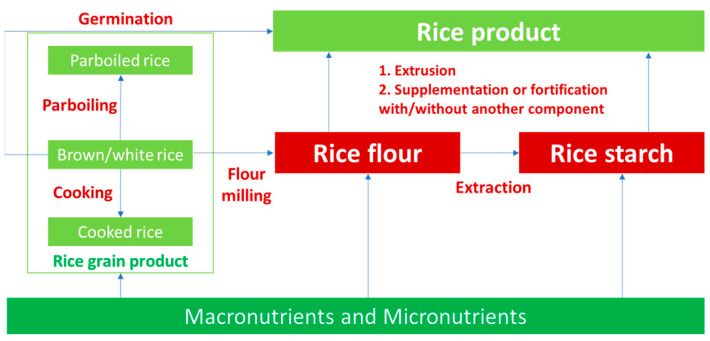
Summarized factor affecting on the glycemic index of rice and rice product.

The most important source of calories for communities around the world is carbohydrates, which have been divided into groups based on how easily they can be digested and how they behave in the digestive system. The overall discussion in this section is outlined in Figure 1. Rice’s composition is the most important affecting factor on the characteristics of rice product, especially digestion behavior, which is the mechanism detailed in Section 3.1, Section 3.2 and Section 3.3. The quality of raw materials could lead to a change in the product’s quality, which also means that the low-GI materials could lead to a reduction in the digestion rate of the product. The recent improvement techniques, such as germination and extrusion in addition to production processing mechanisms and their effects on the starch digestibility of its product, are also discussed (Section 3.4). Storage conditions affecting the GI of the product are deeply reported in Section 3.5.

### 3.1. Rice Starch Composition and Its Structure

Rice was divided into three groups, including classification as waxy (0–2% amylose), very low amylose content (2–12%), low amylose content (12–20%), intermediate amylose content (20–25%), and medium-to-high amylose content (20–33%), based on the ratio of amylose and amylopectin, which are known as the two main compositions of starch [3]. The GI value of rice grain, rice flour, and rice starch is primary influenced by the amylose content; therefore, rice with a high amylose concentration typically has a lower GI value, which can be seen in Table 1. Early studies reported and explained that the high amylose content causes starch to gelatinize incompletely during cooking, whereas amylopectin is completely gelatinized during this process. In addition, the high amylose content also affects the cooking time because a certain amount of energy is required to gelatinize the starch [47]. Recent meta-analysis by Cai et al. [48] showed that less-gelatinized rice starch resulted in a significant reduction in the insulin incremental area under the curve (iAUC), leading to a lower glycemic index. However, starch composition also affected the eating and cooking properties, as well as the functional properties. Thus, the development of novel rice varieties with desirable quality and improved nutritional and functional features may benefit from screening for rice grain and nutritional properties using in vitro starch digestibility tests related to the amylose content and glycemic index.

Restricted gut enzyme access to starch is caused by the formation of complexes between amylose and lipids under various heat-treatment methods [12,20,40]. Amylose–lipid complexes also belong to resistant starch (RS)-type V, which is usually identified by X-ray diffraction, in which the complex gives rise to the V-crystal with peaks at approximately 7°, 13°, and 20° [12]. Resistant starch is also as known as the starch diffraction that resists digestion by human pancreatic amylase in the small intestine and thus reaches the colon to be fermented by gut microbes [49]. Since these lipid–amylose complexes can only be found in conjunction with amylose, rice, and rice products with the highest amylose content, there will be a greater number of these complexes. A recent study showed amylose content in rice grain was negative correlated with estimated GI due to the increasing formation of resistant starch under ultrasound, combined with annealing and chilling [12]. Another study [50] also reported that the different formation level of the amylose–lipid complex was found in parboiled rice due to the different amylose and amylopectin content. There was also a strong correlation between RS, non-resistant starch levels, hydrolysis index, and amylose content in cooked Philippine brown and milled rice [43].

The structure of polysaccharides also affected the ability of resistant starch to the hydrolysis of amylolytic enzymes. The short chain of amylose could promote the reassociation and re-order process to form the new compound which is intolerant of the hydrolysis enzyme [12]. It is well known that amylose has a stronger ability to complex with lipids than amylopectin. However, the long chains of amylopectin could also form the complexes with lipids [50]. The study of Dries et al. [51] provided beyond-state-of-the-art information on the amylose degree of polymerization (DP)-dependent V-type crystal formation for various granular starches, including rice.

### 3.2. Macronutrients

The other two main compositions are protein and lipid, which also greatly affect the digestion rate of rice and rice products. The protein content of rice grain/flour ranged from 4.5% to 15.9%, while the lipid content was much lower, as shown in Table 1. The degree of digestibility of rice depended on the interaction between starch and protein. The interaction between endogenous protein and starch is the main reason, which prohibits the digestive enzyme’s contact to the starch to be hydrolyzed, which means that the protein could act as the inhibition factor [52]. Evidently, the study of Ye et al. [53] showed that the deproteinized rice flour has a faster rate of digestion than the native one. Furthermore, one of the strategies to reduce the glycemic index of Asian foods and meals as purposed by Wee and Henry [54] was adding exogenous proteins from various sources due to the interaction of protein–starch and/or the formation of a network surrounding the starch granular that acts as the protection wall to enzyme accessibility and digestion. The amount of soluble starch and glucose released after digestion is negatively correlated with the amount of protein supplied, which means that protein may form hydrogen bonds with starch granules. By increasing the protein content of brown rice noodles, the glycemic index was reduced [55]. In addition, protein may also control the retrogradation and gelatinization of starch, reducing the digestibility of starch [54]. Another mechanism effect of protein on starch digestibility is that the protein could act as a factor to promote the interaction between starch and lipid. To understand the formation ternary structure at the molecular level, the study of Chao et al. [56] showed that protein could enhance more interaction between amylose and fat molecules. As a result, the molecular structure becomes large and more complex, which also has a more short-range ordered structure, a helical structure, crystals, and a lower starch digestibility. The similar results about the formation of the starch–lipid–protein ternary structure are also reported by Chen et al. [57]. Furthermore, the endogenous proteins in Indica rice cultivars lower the glycemic index and decrease starch swelling to cooperate with the presence of lipid, likely by producing a coating around the starch granule that prevented swelling and inhibited the action of digestive enzymes [53]. Furthermore, protein and the enzyme that breaks down starch may interact, reducing its enzyme activity and the ability to break down starch [57].

Lipids and amylose have long been understood to produce inclusion compounds, with the hydrocarbon part of the lipid residing inside the helical cavity of amylose. These complexes’ slow digestion has been linked to decreased postprandial insulin and blood glucose levels [22]. Firstly, rice with a greater amylose content may have a lower GI due to type 5 RS (amylose–lipid complex), which may be a contributing factor (Section 3.1). Secondly, endogenous lipids in rice grain could reduce its starch digestion rate. According to [13], lipids are mainly found in the embryo and aleuronic layer of rice, which can remarkably decrease the GI. Furthermore, [13] also reported that white rice flour with the presence of lipids could reduce the GI due to the complexation mechanism. Rice flour without endogenous lipids observed a significantly higher rate of digestion than its native rice flour [53]. Thirdly, the structure and type of fat molecules could affect the production of the starch–lipid complex. Zheng et al. [58] also purposed that the shorter chain and lower-molecular-weight unsaturated fatty acids could promote a more ternary structure (starch–protein–fatty acid), which lowers in vitro enzyme digestibility. Starch could also interact effectively with 12-carbon fatty acids by the nano-complexation process, which produced a higher level of resistant starch and slow digestive starch [59]. Long-chain saturated monoglycerides are more resistant to enzymatic digestion than short-chain (saturated and unsaturated) monoglycerides. Therefore, when complexed with starch molecule, they might also lead to a lessened blood glucose response according to research by Farooq et al. [60]. During the food processing, the interaction between fat and starch molecule also occurred, which affected on the characteristics of the product, as well as its digestibility [61]. With the presence of high lipid content in rice pancakes, the estimated glycemic index was at the medium–low level (60.8) [41]. The collapsed helical structure of the starch–lipid complex makes them highly resistant to enzymatic hydrolysis due to the prevention of the enzymes, making them less accessible for binding to amylose molecules [62]. The accessibility of the enzyme to hydrolyze the starch is also decreased by the starch–lipid complex, which increases the entanglement of resistant starch between amylose and amylopectin [61].

### 3.3. Polyphenols and Dietary Fiber

In recent years, there has been increasing interest in the effects of various polyphenols on the in vitro digestibility of rice starch, rice flour, and rice products. Polyphenols could not only act as inhibitors of enzyme hydrolysis but could also interact with starch to form starch–polyphenols complexes [11,63,64]. Recently, the review of Ngo et al. [11] showed that the complexation of starch with various polyphenols could promote the formation of V-type crystalline structures, as well as resistant starch and slowly digestive starch, including rice starch. The formation of binding between the starch and polyphenols by electrostatic interactions (i.e., hydrogen bonds), as well as *van der Waals forces*, lead to a reduction in the hydrolysis rate of rice starch [11]. Furthermore, amylolytic enzyme inhibition was found during the digestion process of starch–phenolic complexes, which modulates the glucose release rate. This was supported by the study of Aalim et al. [65], which proved that endogenous phenolic compounds are more critical factors compared to dietary fiber for retarding in vitro starch digestibility of starch-based foods prepared with black rice, and their processing is favorable for the dietary management of metabolic disorders. Supplementing the polyphenol from butterfly pea flowers also reduced the in vitro starch digestibility of cooked rice with the acceptance of the consumer [66]. A high negative correlation between the GI and the total phenolic compound in rice was found in the study of [39]. Recently, the study of Kim et al. [67] also showed a possibly reduced GI of food by polyphenol both in vitro and *in vivo*. In addition, the most interesting, in recent years, is colored rice, known as pigmented rice, which is also considered to have a promisingly low GI. When comparing the GI of white rice and colored rice, due to the high number of antioxidant compounds as endogenous phenolic compounds, the observed GI and GL were lower in pigmented rice [39]. Riceberry is one kind of purple rice cultivar that also showed the potential of low-GI product, which is studied by Raungrusmee et al. [68]. The colored rice varieties not only have the potential to modulate the digestion behavior, but also to become the promising gluten-free ingredient [69]. Therefore, it might be concluded that the polyphenols, intrinsic or extrinsic, have the potential to reduce the digestion rate of rice products.

Another compound that affects the glycemic index of rice, and its product is dietary fiber. Dietary fiber (DF), principally the non-starch polysaccharides of the plant cell wall, is an important component of our diet [70]. DF is a fraction of plant material that resists digestion by the hydrolytic enzymes present in the human gut. Food contained high DF and RS, which could possibly have a low index of digestion [71,72,73]. The recommendation of DF is 38 g/day for men and 25 g/d for women [70]. The outer layer of brown rice contains high amounts of dietary fiber [74], which also contributed to modulating the glycemic index of rice and its flour [75,76,77].

### 3.4. Production and Processing Techniques—Possible Mechanism Effects

#### 3.4.1. Rice Grain

Quality characteristics and the glycemic index of rice could enhance and control by the parboiling process, which is known as a hydrothermal process. The parboiling process led to starch swelling and gelatinizing, the denaturation of the protein structure, and the formation of the lipid–amylose complex in rice [78]. The main process of parboiling includes soaking, steaming, drying, and removing hull (if required) [78]. The gelatinization process occurs when the grain is hydrated with enough water during soaking and heated by steaming. The leaching of some constituents into the soaking medium and the transferring of nutrients within the rice grain occurred during this process [79]. Then, the retrogradation of rice grain was taken after steaming and continued until milling, which also led the starch becoming more difficult to digest [78]. The link between the GI of parboiled rice and process conditions has been recently discussed by various researchers. The formation of amylose–lipid, decrease in crystallinity, and increase in RS content were the main occurring process that led to the lower starch digestibility of parboiled rice [27,79,80]. However, the increase in the degree of starch digestion also occurred due to the insufficient time between the gelatinization and drying stages that result in incomplete retrogradation of starch [13], as well as the effect of drying temperature [15,50]. After parboiling, rice starch still contained substantial levels of double helices. The long-range organized structure of rice starch was broken up by parboiling. The development of V-type crystals in rice was accelerated by high-temperature steaming. The glycemic index of rice is dramatically lowered to medium level by parboiling [15]. Therefore, the effect of parboiling conditions and other drying methods used during industry-scale processing of rice must be studied in detail. These would be of particular interest for value-added products including quick-cooking rice and other ready-to-cook rice-based foods.

The production of graded and polished white rice from harvested paddy is a part of post-harvest paddy processing, which was additionally known as dehulling. Dehulling involves separating several components, including brown rice, hull, and dehulled rice. The dehulled paddy is subsequently milled or polished to produce raw white rice (head rice and broken). The ideal milling operation will yield varied amounts of hull (20%), bran (8–12%), milled rice, or white rice (68–72%), depending on the type of rice and the level of processing. For later usage, this is then packaged and stored. Rice is usually eaten as milled grains that have had the bran layers (pericarp, testa, and aleurone layer) removed. This type of rice is known as white or polished rice. This polished rice has superior cooking and eating qualities compared to brown rice [17]. The rice starch changed its functional characteristics and promoted a higher hydrolysis rate due to the change in size and swelling during the milling process [43]. The loss of nutrients was also one of the reasons that led to a change in the degree of starch digestibility [43]. Therefore, the control of the degree of the milling process is an important consideration for future work.

In brown rice, the non-starchy polysaccharides that make up the bran and cell wall of the endosperm serve as a barrier to the starchy endosperm. Any instability to the intact bran layer, which is removed during polishing, can enhance the rate of starch digestibility because digestive enzymes can more easily access the starchy content [75]. Various studies reported that white rice has a greater GI than brown rice, which also greatly depends on the degree of polishing [17,44]. The relationship between dietary fiber, antioxidant activity, and the GI of polished rice was also reported by proving the importance of the polishing degree on the quality of rice grain, as well as their flour for further processing.

For human consumption, rice is cooked. During that process, the starch of the grain gelatinizes, giving the finished product desirable textural qualities. Additionally, cooking can increase the bioavailability of many nutrients. Different methods were applied to cooking rice as conventional methods, including pressure cooking, cooking in electric cookers, or using microwave or stir-frying, which led to differences in the quality and characteristics of cooked rice [81]. In general, heating starchy meals can cause starch granules to gelatinize, losing their amylose and amylopectin crystallinity and creating a disordered structure that makes starch more prone to enzymatic hydrolysis [82]. While gelatinized starch and the leaching of starch molecules due to high cooking temperature in cooked rice led to higher starch digestion rate [66], the complexation between rice starch and lipids, as well as starch granular, was coated by lipid-reducing the GI of stir-fried rice [83]. Differences in cooking temperature and time and the ratio of rice to water also modulated the GI of cooked rice. For instance, for the production of instant rice, ref. [84] optimized the cooking temperature and water to rice ratio. They found that cooking rice at a lower temperature for a shorter time while using a higher water ratio could lower the GI of the grain because the rice’s structure had fewer voids and a more compact periphery, which limited enzymatic digestion. Microwave cooking also reduced the GI of cooked rice [85], and the new technology as a retort also applied to cook rice (121 °C, 30 min, 15 psi) and showed the effectiveness of reducing the GI of cooked rice [9]. However, further study should be considered and optimized.

#### 3.4.2. Rice Flour

Flour milling, known as the size-reduction process, is a crucial step to receive the fine flour for applying in food products, such as bread, cake, cookies, etc. The particle size distribution in rice flour has greatly influenced the physicochemical properties and the digestion rate of rice flour in both in vitro and *in vivo* studies [81,86]. The smaller particle size is more likely to exhibit an increase in the rate of digestibility due to the increase in the surface area to make contact with amylolytic enzymes. Given that the increase in the surface area is also associated with the rice flour’s swelling power, solubility, and gelatinization temperature, all of which in turn related to the digestibility [86]. It could be seen that the particle size is the main impact on the GI of rice flour; however, it is also affected by the initial sources. For instance, Lee et al. [74] examined how quickly the Japonica and Indica rice cultivars digest starch. The Indica rice maintained its compact structure throughout the simulation of in vitro gastrointestinal digestion up until the middle phase of small intestinal digestion, which led to a slower rate of starch hydrolysis than in the case of the Japonica type. Indica rice also contained a larger concentration of the lipid–amylose complex, which could account for the slower starch digestion.

#### 3.4.3. Rice Starch

In general, rice starch was extracted from its flour. The most notable difference between rice starch and rice flour is the contents of protein and lipid. Industrial products and functional foods frequently contain rice starch. Starch is mostly separated from protein, fiber, and fat during the isolation process. Thus, it is crucial to avoid amylolytic or mechanical damage to the starch granules, deproteinize the starch effectively, minimize the loss of tiny granules, and prevent starch gelatinization [87]. The alkaline process is usually used for the isolation of rice starch with/without a physically assisted method as ultrasound, freeze–thaw infusion method [87]. However, the link between the GI of rice starch with the extraction method is still limited. Mostly, the modification was applied to reduce the GI or characteristics after isolating rice starch [87].

#### 3.4.4. Rice Products

Rice value-added products and processed rice are the most important parts in the food industry. A recent report showed that 10% of the total amount of rice available is turned into various value-added products, in addition to being consumed as whole rice [78]. In this sub-section, the mechanism of the most recent significant techniques affect the digestion characteristics of rice product, as extruded rice and germinated rice are detailed. Furthermore, recent publications about the influences of ingredients (rice starch/flour and others) and processing condition on the starch digestibility of bread, noodles, and cookies are also basically provided and discussed.

Extrusion technology was applied to produce various rice products, such as modified starch-based snacks, precooked breakfast cereals, snacks developed from a variety of cereal blends, vegetable-based foods, etc. The main step of extrusion process concludes mixing, heating, cooking, sharing, and shaping, which have an effect on the digestion rate of the product [22,88]. Rice extrusion causes protein denaturation, amylose–lipid complex formation, starch polymer molecular fragmentation, and partial or total breakdown of rice’s crystalline starch structure [22]. Compared to milled rice or extruded wheat products, eating extruded rice can lower the GI. This can be ascribed to the process’ gelatinization and retrogradation of starch. Additionally, three theories have been considered for the decline in starch digestibility following the extrusion procedure: (i) the formation of covalent cross-links of other molecules; (ii) starch could be coated by other molecules, such as protein and lipids, to prevent amylase digestion; and (iii) the formation of a complex structure, such as RS, amylose–pectin complexes, and starch–phenolics complexes [89]. The change in crystalline patterns in extruded rice products could also lower the glycemic index [22].

One of the low-cost techniques for preparing rice that can enhance its nutritional value is germination. Although brown rice has a relatively high nutritional content, it is not commonly consumed since it is challenging to prepare and indigest and has poor textural qualities in addition to difficulties with long-term preservation [14]. GABA, phytic acid, tocotrienols, potassium, zinc, γ-oryzanol, and ferulic acid are micronutrients found in the germinated brown rice, most of which are simple to digest and absorb [14,90]. Consuming brown rice that has been germinated has several benefits for those with diabetes. Due to the high dietary fiber content and bioactive substances that inhibit the ability of the digestion of α-amylase in the small intestine, consumption of germinated brown rice decreases the postprandial blood glucose response in healthy subjects [90]. In addition, the decrease in starch content and loss of the A-type pattern of the crystal structure were found during the gemination process [74]. However, it is also limited to the literature reporting on the starch digestibility of germinated rice. Recent reporting showed that rice that was created through germination had less of an impact on the bioactive compounds, including more dietary fiber, and had a low–medium GI [29]. More evidence should be developed to show the link between digestion characteristics and germinated rice.

A significant amount of dietary carbohydrates and energy is found in cereals, such as rice, which is consumed as a staple food by a vast population worldwide. The glycemic index of rice and rice products may also be categorized as medium–high. In most cases, rice is consumed with other food items in steamed or boiled form (pulses, legumes, vegetables, nuts, vegetable oils, meat, and seafood). Due to the increased protein, fiber, and fat from other foods in the mixed meal, it is expected that the GI of rice would differ from that of only rice consumed. Recently, some research showed the effects of the mixed meal on postprandial hyperglycemia [91]. Fortification or supplementing low-GI ingredients during formulation or processing could reduce the GI due to the formation of enzymatic tolerance compounds or delaying gastric emptying [3]. The formation of a physical barrier between starch and amylolytic enzyme by supplemented ingredients leading to a lower GI value in cooked rice, rice noodles, rice meals, etc. was also reported [65,92,93]. The best options for diabetics would be food combinations with a low GI rating because they are linked to lower glucose responses; however, the appropriate formulation or recipe should be considered to not only improve the nutritional and health benefits but also without a change in the sensory value of the product.

### 3.5. Storage and Retrogradation

Retrogradation is a phenomenon of starch gel. Following cooling and gelatinization, the molecules of amylose and amylopectin reassemble to produce a more organized structure. Due to the nutritional importance of this process, which involves the release of glucose into the bloodstream gradually from retrograded starch, it is less susceptible to enzymatic digestion [94]. Several physical modifications, such as an increase in viscosity and turbidity, take place inside starch granules during the retrogradation process. The formation of type-3 resistant double helices occurred during the retrogradation process, and the degree of retrogradation is typically inversely linked with the starch digestibility [94]. Storage time and temperature are the most important factor affecting the degree of retrogradation, as well as the GI of rice product [3].

## 4. Recent Technologies to Reduce Glycemic Index of Rice and Rice-Based Products

Rice is a staple food in most Asian countries, which is also considered as a main material in numerous products, such as rice cakes, rice noodles, prepared breakfast porridge, and baby food. Because of the adverse effects of high-GI foods, the modification method was developed to fulfill consumer requirements. However, depending on the materials or food products, the method and effectiveness of the technique are different.

### 4.1. Rice Grain

Recently, most studies have shown that brown rice or colored rice have a lower glycemic index than white rice due to its nutritional profiles and functional properties. The degree of polishing and milling could vary the glycemic index of rice. For instance, the GI of Sinandomeng brown rice (Philippines) was lesser, by approximately 26%, than milled rice [44]. Another study [75] on Indian rice also reported that white rice had the highest GI (79.6), followed by undermilled rice (73, −8.29%) and brown rice (57.6, −26.64%). Furthermore, the colored rice (Riceberry) also showed a lower GI, by about 24.9%, than white rice (Hommali) [42]. The polyphenol-rich sources in colored rice and the presence of lipid and/or protein in the outer part of brown rice could lead to a decrease in the digestion rate [42]. This was also supported by Somaratne et al. [17], who showed that GI had an inverse association with red basmati rice with increased antioxidant activity. After polishing, 100% polished rice had less antioxidant properties and a higher digestion rate than 10% polished rice. Therefore, the glycemic index could be controlled by process operation parameters.

Various methods were also applied to reduce the glycemic index of raw rice (uncooked form) as shown in Table 2. The percentage of reducing GI was found to be around 8–21% compared to the native form due to different operation conditions. The parboiling technique was the technique that was the most applied to reduce the GI of rice in recent years. Recently, some improvements in parboiling conditions, such as parboiling pressure or combinations with other methods, also conducted and showed the effectiveness to reduce the digestion rate of rice. By deeply investigating the effect of the processing conditions, most of the research shows that designing less digestible parboiled rice in the future is valuable, which may enlarge the manufacturing of parboiled rice [15]. Some emerging technologies, such as sonicating, chilling, and irradiated treatment, also significantly reduced the GI of rice, which are shown in Table 2. These techniques are considered green and sustainable technologies due to their lack of an impact on the environment and because they are chemical-free. However, the organoleptic properties of rice should be further considered. The combination of two or more methods might also reduce the GI, as well as maintain the nutritional and sensory properties of rice grain.

Given the popularity of cooked white rice as a staple diet, finding the methods to reduce its starch digestibility is a key point for the diabetic population. Recently, Shen et al. [18] found that pre-soaking before cooking could reduce the digestion rate of cooked rice. Cooking rice with lipids is another efficient strategy to lower GI. It was shown that after heating rice with palm oil, the development of an amylose–lipid complex decreased the in vitro digestibility of non-waxy rice [60]. The cooking methods also affected the digestibility of cooked rice. The RS content of cooked rice was found to follow the following order: microwave > electric cooker > stone pot > autoclave in the research of Lee et al. [82]. Cooking rice with a natural polyphenol-rich pigment could potentially reduce starch digestibility due to the increase in the RS. The research of Chusak et al. [66] reported that cooking rice with butterfly pea flower could increase the RS content by double. This finding supports the future direction of cooperation between various sources of polyphenol in developing low-GI cooked rice or starchy foods.

**Table 2:** Current techniques to reduce the glycemic index of rice.

Applied Technology	Findings on Starch Digestion Behaviors	Possible Mechanisms	Ref.
Parboiling with different steaming conditions	Different pressure and time of steaming led to reduction in the GI of rice. Steaming at 1.5 kg/cm^2^ for 20 min was found to be more appropriate for lowering GI (~47) of Pusa Basamti 1121 rice.	The change in nutritional profiles and multi-scale structure of rice grain led to vary the GI of parboiled rice under different steaming conditions.	[95]
Parboiling, polishing 10%, parboiling plus polishing	The kinetic digestion rate constant (*k*) of 10% polished rice, brown rice, parboiled rice, and parboiled-polished rice were 3.68, 3.25, 3.9, 2.49 (×10^−2^ min^−1^), respectively.	The presence of the aleurone layer and pericarp on the surface of parboiled rice might act as barriers to enzymes and limit the starch hydrolysis.	[96]
Parboiling with different soaking conditions	Parboiled rice had lower GI than non-parboiled rice. A significant reduction in GI was found in glutinous rice. Soaking with 0.2% acetic acid led to more decreased digestion rate of treated rice than soaking with medium with/without NaCl.	The two main changes that occurred during parboiling are gelatinization and recrystallization, which could result in increased quantities of resistant starch and reduced GI. Starch–protein interaction might have limitation in the starch’s ability to be digested. Saline medium might have raised postprandial plasma glucose by promoting amylase activity and accelerating the digestion of starch.	[80]
Parboiling and heat-moisture treatment	Pressure cooking and heat-moisture treatment after parboiling rice could reduce about 10% GI compared to un-treated sample.	The increase in RS and SDS after treatment led to a reduction in the rate of digestion.	[97]
Ultrasound (15/30 min, 40–100% amplitude, 665 W) and chilling (4 °C, 24 h) on KMDL105 rice and CN1 rice	Sonicated modification increased approximately 11.96% eGI of KMDL105 rice compared with native sample, while ultrasound-chilled treatment on rice showed a reduced pattern. The treated CN1 rice had lower eGI (~8%) than its native when modified by either ultrasound or ultrasound-chilled method.	Ultrasound modification affects the starch crystalline structure. However, the combination between ultrasound and chilling led to rearrangement of starch molecules that help lower eGI.	[20]
Open steaming and pressure parboiling with different rice varieties	In terms of GI, non-parboiled rice varied from 76.88 to 83.37. Rice that was parboiled had a lower GI than rice that was not parboiled (between 75.54 and 79.90 for pressure parboiling and between 77.02 and 80.37 for open steaming parboiling).	The increase in RS during parboiling process led to a decrease in the GI of rice. Amylose content and hardness of rice also inversely affect digestion rate. Complexation between amylose and lipid also might occur and change digestion behaviors.	[98]
Brown rice, parboiled brown rice, germinated parboiled brown rice, and polished rice	The analysis GI of rice using the method of International Standard Organization was carried out in the following order: polished rice (83.10 ± 5.10) > brown rice (66.21 ± 7.78) > germinated parboiled brown rice (60.58 ± 6.48) > parboiled brown rice (50.10 ± 5.37).	In the high quantity of dietary fiber in brown rice, parboiled brown rice, and germinated parboiled brown rice, the rate of digestion may be slowed down. In addition, the starch structure, amount of amylose, dietary fiber, and bioactive substances may all play a role.	[29]
Gamma irradiation (5 kGy and 10 kGy) using Cobalt-60 gamma irradiator	An increase in amylose content was found when rice grain was treated with 10 kGy irradiation (+42.8%). The GI of unirradiated, 5 kGy, and 10 kGy rice were 75.02, 68.35 (−8.89%), and 66.51 (−11.34%), respectively, found using an *in vivo* starch digestibility study on animals.	Gamma radiation modifies the structure in both amorphous and crystal regions, leading to the splitting or deformation of glycosidic bonds.	[34]
Parboiling with different rice genotypes	An approximate 10% reduction in GI was found when applying the parboiled treatments.	Higher gelatinization levels in parboiled rice may raise the RS and lower the GI. Gelatinized starch generates type-III RS after parboiling, which slows starch digestion.	[16]
Parboiling with various conditions	A 20.9% GI reduction in parboiled rice compared with untreated rice was found when the paddy was soaked at 75 °C for 4 h.	Parboiling technique could make the smooth morphology, formation of V-type crystals, and a tight internal structure, which might reduce the digestion rate.	[15]

### 4.2. Rice Flour and Rice Starch

Rice flour (also rice powder) is a form of flour made from finely milled rice, while the rice starch was extracted from rice flour using the common method, also known as alkaline extraction. The endogenous compositions of rice flour/starch made up their GI, which is discussed in Section 3. Despite having a protein level that was around ten times higher than that of lipids, rice flour without lipids had a little lower starch digestibility than rice flour without proteins [53]. In addition, the particle size is one of the most influential on the GI of rice flour/starch. For instance, the study of Farooq et al. [86] found that the digestion rate and extent were markedly reduced with the increasing particle size of four milled rice (waxy, white, black, and brown rice).

Nowadays, physical modification is the trending method due to growing consumer health issues. Physical treatments are not only considered chemical-free methods, but they also show effectiveness in reducing the GI of rice flour/starch [99]. Innovative physical modification was widely applied to modulate the GI of rice flour and starch, such as microwave treatment, high-pressure homogenization, irradiation, and ultrasound, as shown in Table 3. Recently, reducing the glycemic index in rice starch and flour by endogenous and/or supplemented polyphenol was found [11]. Polyphenols act as inhibition factors of enzyme hydrolysis, which is presented in many kinds of sources and studied by many researchers [100,101,102,103]. Furthermore, polyphenols could bind with other molecules to produce tolerant enzymatic components. Polyphenols could reduce the activity of glucose transporters inside the human body, which also lower the peak of blood glucose in the body [88]. It could be seen that researchers have been more interested in novel modification techniques, along with sustainability criteria. These techniques have composite advantages, including quick treatment periods, non-thermal and economical operations, non-toxic effects, low water use, and scale-up processes. They could also be combined to the low GI of rice flour/starch in further study.

**Table 3:** The impact of various modifications on rice flour/starch digestibility.

Material Types	Modification Methods	Main Finding	Possible Mechanisms	Ref.
Broken rice flour (Haowang Co., Rui’an, China)	Co-extrusion with grape seed proanthocyanidins (GSPAs)	A significantly lower equilibrium hydrolysis and k constant were observed in extruded rice flour with grape seed proanthocyanidins.	The rice grains undergo progressive degradation, resulting in a macroscopic change in which the digestive solution assumes a pink coloration. The GSPAs that have been released may have the potential to impede the activity of digestive enzymes, hence contributing to a reduction in starch hydrolysis.	[104]
Broken rice flour (Haowang Co., China)	Co-extrusion with chlorogenic acid (CA)	The RDS decreased markedly (down to 41.28 ± 2.66%), and there was a pronounced increase in RS, which varied from 10.28 ± 2.62 to 31.16 ± 0.50%.	The presence of hydroxyl groups in CA enables the formation of hydrogen bonds with the hydrogen groups of α-D-glucose molecules found in amylose or amylopectin chains inside rice starch. This interaction potentially hinders the formation of α-1,4-glycosidic and α-1,6-glycosidic bonds, thereby affecting the binding sites of digestive enzymes involved in carbohydrate breakdown.	[105]
CN1 and KDML105 rice four	Heat-moisture treatment (HMT 30%, 110 °C)	Heat-moisture treatment could reduce approximately 23% eGI of rice flour (both CN1 and KDML105).	The application of HMT has the potential to enhance the intermolecular connection of starch chains, hence strengthening the bonding between starch granules and leading to the formation of a more organized and structured system. The application of HMT has the potential to facilitate the production of amylose–amylose and/or amylose–lipid complexes, hence inducing robust interconnections among starch granules.	[40]
Indica, Japonica, and waxy rice flour	Radio frequency treatment	The resistant starch content of Indica, Japonica, and waxy rice flour increased from 39.04% to 42.02%, from 39.95% to 45.12%, and from 36.26% to 43.61%, respectively.	The use of RF treatment led to the formation of intermolecular aggregates through the interactions between starch granules, proteins, and lipids, thus leading to an elevation in the levels of SDS and RS.	[106]
High amylose rice flour [Dodamssal (DDS) and Ilmi (IM) cultivar]	Steaming, roasting, and steaming plus roasting treatment	Roasted DDS rice flour and steamed–roasted DDS have the lowest level of starch hydrolysis. Steaming plus roasting could greatly reduce the eGI of both cultivars.	The process of gelatinization of starch is facilitated by the presence of moisture and heat. The heat processing of starch has the potential to induce a substantial increase in the rate of starch hydrolysis. The amylose content of starch is a crucial determinant of its digestibility as it influences the production of complex molecules.	[107]
Rice flour (3 to 30% moisture content)	Microwave treatment (MWT)	The samples treated at 20% and 30% moisture showed 40% and 47% RDS, 48% and 70% lower SDS, and 90% lower RS than the untreated flour.	The MWT facilitated the development of protein structures, such as random coil, α-helix, and β-turn. The utilization of MW treatment facilitated the alteration of the structural and thermal properties of rice flour, hence affecting its rate of starch hydrolysis.	[21]
Rice starch (23.5% amylose)	Complexation with fatty acid by high-pressure homogenization (HPH)	When rice starch was complexed with fatty acids under HPH, its digestibility was drastically reduced.	The formation of complexes between rice starch and fatty acids was achieved through the process of high-pressure homogenization. A compound was formed in the shape of V-type crystals, resulting in the induction of an ordered structure. The digestibility of rice starch was seen to undergo a considerable reduction following its complexation with fatty acids.	[108]
Rice starch (Jinnong Biotechnology Co., Ltd., Wuxi, China)	Complexation with unsaturated fatty acid by HPH	Under HPH, the complex of rice starch and unsaturated fatty acids (R-UFA) was produced. Lower digestion of R-UFA was caused by longer unsaturated fatty acid chains.	The incorporation of unsaturated fatty acids has the potential to modify the configuration of starch molecules, enhance the formation of aggregates, and thus impact the digestibility of starch. Under the treatment of HPH, unsaturated fatty acids undergo complexation with rice starch, resulting in the formation of a single helix by hydrophobic interactions. The compound underwent a process of induced ordering, resulting in the formation of a V-type crystalline structure. The increased length of the carbon chain in unsaturated fatty acids resulted in a decrease in the digestion of these fatty acids.	[109]
Rice starch (S7260, Sigma, St. Louis, MO, USA)	Co-extrusion with and Chinese berry leaves polyphenol (CBLPs)	A reduction of about 32.6% RDS was found when the complexation between rice starch and Chinese berry leaves occurred.	Pores within the interior part of the kernel and a loosely arranged structure were found through SEM analysis, which facilitated the action of enzymes in breaking down starch through hydrolysis. Additionally, they enhanced the subsequent release of CBLPs throughout the process of digestion. Meanwhile, CBLPs have the potential to effectively suppress the enzymatic activity of α-amylase and α-glucosidase. The increase in RS content of restructured rice can be attributed to the combined influence of rice structure and the inhibitory impact of CBLPs.	[88]
Rice starch (14.8% amylose)	Extrusion with several types of fatty acid	Extrusion of rice starch and linoleic acid have the highest RS (15.7%) and lowest eGI (88.4) compared with other samples.	Complexes containing fatty acids with shorter carbon chains have greater heat stability. The complexity of content is observed to grow with a decrease in the number of carbons and the degree of saturation of fatty acids. The combination of amylose, amylopectin, and unsaturated fatty acid results in the formation of a structure that is resistant to enzymatic degradation.	[22]
Rice starch (Wuchang, China)	Microwave (MW) and cold plasma treatments (CP)	The dual-modified rice starch has a higher RS. Dual modification could reduce the digestion rate than single modification.	RS content of rice starch exhibited an increase after the application of MW and CP. The utilization of MW and CP treatments, which resulted in the polymerization of amylose molecules in rice starch, leading to the formation of bigger molecules. The co-treatment impacted the relationships between amylose molecules, amylopectin molecules, amylose–amylopectin chains, and the enzymatic susceptibility of the starch double helix.	[110]

### 4.3. Rice Product

Rice can produce a variety of products, such as porridge, bread, noodles, or cakes. In recent years, scientific researchers have also been developing recipes, as well as suitable preservation and processing methods to create products with high sensory quality that can reduce the GI of the product. By various combinations of different ingredients, as well as the appropriate operation method, low-to-medium GI products have been created, as shown in Table 4. The source of the raw material strongly influences the digestibility of rice products. Firstly, low-GI rice flour or rice starch could reduce the starch digestibility of the product. Secondly, additional ingredients, such as those rich in proteins, lipids, or polyphenols, could form complexes such as starch–protein, starch–lipid, starch–polyphenol, or tertiary and quaternary complex structures. Finally, the formation of resistant starch might also occur during the processing of rice products when combining various kinds of powder or ingredients. Therefore, the future trend might find suitable materials and innovative scientific processing techniques to process rice-based products that have both sensory and economic value and can meet the health needs in low-GI rice products for customers.

**Table 4:** Recent developments in low-GI products from rice.

Product Types	Formulation/Applying Method	Key Findings	Possible Mechanisms	Ref.
Rice bread	Hommali rice flour (HM, white rice) and Riceberry rice flour (RB, colored rice) were used for bread processing.	The eGI value of RB was lower (37.4%) than that of HM bread.	The low pGI value of rice bread may be linked to several factors, including the presence of anthocyanin in the formation of complexes and inhibition factor, greater granule size, and the presence of substances, such as SDS and RS.	[42]
	Okara flour (0–21%) was added to study their effect on starch digestibility of steamed rice bread (SRB).	The eGI of SRB decreased from 79.14 to 74.17–68.91 because of the addition of Okara, which dramatically enhanced the amylose content, SDS, and RS of SRB.	The digestibility of SRB may be reduced due to the presence of certain non-starchy substances on the surface of Okara. These substances, including dietary fiber, protein, and lipids, become attached to starch particles during the milling and gelatinization processes. As a result, the diffusion of enzymes into the starch gel structure is hindered, and the adsorption site of the substrate is blocked. This ultimately leads to a decrease in the digestibility of SRB.	[111]
	Gluten-free bread made from rice flour was supplemented with hydroxypropyl methylcellulose (HPMC), whey protein concentrate, and soy protein isolate (4%).	The eGI of bread decreased from 84.08 to the range of 67.20–71.04 due to the heteropolymer structure of HPMC and protein-covered starch granules.	The digestibility of starch in gluten-free bread was primarily diminished by the inclusion of a combination of HPMC, proteins, and butter in the dough formulation. The use of HPMC and whey protein additionally enhanced the enhancement of antioxidant activity, as assessed by the DPPH experiment.	[112]
Instant rice	White rice with different amylose content was cooked at different conditions [cooking temperature (82–90 °C), water: rice ratio (1.0–1.9-fold)].	The optimal cooking condition for producing lower eGI instant rice was 82 °C with 1.9-fold water volume in all the three Thai rice cultivars evaluated.	The observed decline in eGI may be attributed to the elevated levels of SDS and RS, which can be attributed to the reduced enzymatic accessibility of whole rice kernels when cooked with greater water quantities at lower temperatures.	[84]
Rice dumpling	Dumpling was prepared with native rice flour (KDML105 and CN1) with substitution of heat-moisture-treated rice flour.	The eGI of dumplings from non-treated KDML105 and CN1 flour were 80.76 and 73.48, respectively. Dumplings made from treated flour had significantly lower eGI (appr. 10%) than those made from their native flour.	The utilization of HMT-modified rice flour leads to enhanced intermolecular interactions among starch molecules, potentially accounting for the altered digestibility of rice starch and thus contributing to a reduction in the digestibility of the dumpling.	[40]
Rice cookies	Gluten-free cookies were made using rice flour with a different ratio of carboxymethyl cellulose (CMC) and baking conditions.	Resistant starch, eGI, and GL were recorded as 7.20%, 44.60 and 17.51, respectively, in final cookies when cookies were produced under optimal conditions (temperature of 185 °C, a baking time of 22 min, CMC of 0.8%).	Longer time of baking boosts retrogradation during the chilling process and restricts the rate at which starch is hydrolyzed. The birefringence of starch undergoes partial loss, resulting in a modification of its digestibility. This alteration can be attributed to the disruption of native bonds and subsequent development of resistant bonds. The adverse associations between CMC and perceived general eGI and GL can be ascribed to the interactions among CMC, starch, and fat that occur in cookies during the baking process. Furthermore, it has been observed that the molecular structure of amylose, characterized by linear and tightly packed chains, can result in a reduction in the accessibility of catabolic digestive enzymes.	[113]
Rice cake	Three rice flours (Indica, Japonica, and glutinous) and low-gluten wheat flour were applied to prepare rice cake in chiffon style	Rice cakes have a GI of 46.91, 51.81, 61.63, and 61.35 when made from wheat, Indica, Japonica, and glutinous flour, respectively.	The reduced digestibility can be attributed to factors beyond the amylose/amylopectin ratio, including the presence of additional components such as protein and fat. The occurrence of the formation of new chemicals is possible.	[114]
	Rice cake was prepared from low-GI rice flour subjected to starch hydrolyzing enzymes (0.04% α-amylase and 0.07% glucoamylase).	GI of rice cake was 43.76, and thus comes in the low-GI category of foods. Sensory score of rice cakes was acceptable, though lower than control (using normal rice flour).	The observed decrease in the GI of cakes can be attributed to the increased presence of RS content during the baking process. The process of baking not only results in a reduction in the amount of starch that is resistant to digestion, but it also decreases the pace at which the non-resistant starch component is digested.	[115]
	Matcha (MAT), tea polyphenols (TP), and catechin (CAT) were used to investigate the effect on starch digestibility of rice cakes during storage under different temperatures.	Polyphenolics, particularly CAT, prevented rice cakes’ starch from being digested, which simultaneously raised the quantity of RS.	The primary effect of polyphenolic compounds, specifically TP and CAT, was the inhibition of starch retrogradation in rice cakes. This inhibition occurred through the interaction between the polyphenolic compounds and the starch chains, resulting in a reduction in the amount of bound water. This reduction was evident by observations of water migration and a decrease in relative crystallinity. The observed decrease in hardness and rise in adhesiveness can be attributed to the inhibitory effect of polyphenolic chemicals on the cross-linking of starch chains.	[106]
	Steamed rice cakes were formulated by mixing various ratio of Riceberry flour, xanthan gum, and glutinous rice flour and filling them with red bean paste.	The optimum steamed rice cakes stuffed with the red bean paste used isomaltulose as a sucrose replacer and were classified as the medium GI food.	There was a negative correlation seen between the apparent amylose content present in raw rice starches and their digestibility. An increased amylose concentration typically led to a more orderly molecular arrangement within the structure, as well as a higher degree of crystallinity, rendering it less susceptible to enzymatic digestion.	[116]
Rice porridge	White kidney bean extract (WKBE) was mixed with rice porridge to investigate the change in eGI.	The hydrolysis of rice porridge’s starch might be reduced by WKBE. Adding 43.2 U/g WKBE caused the product’s eGI to drop from 85.18 to 45.01.	The GI of porridge exhibits inherent differences due to changes in starch composition. However, the utilization of white kidney bean extracts has been found to successfully restrict the hydrolysis process of diverse starches, thereby leading to a lower glycemic index.	[117]
Rice noodle	RD 31-native autoclaved resistant starch (RD 31-NARS), xanthan gum (XG), inulin, and defatted rice bran were used to formulate low-GI gluten-free noodles.	RD31-NARS could be used for formulation of low-GI noodles with inulin and defatted rice bran. The final product [RD31-NARS + XG (2.5%) + Rice bran (5%)] has an eGI of 48.01	Inulin functions by decreasing the glycemic index value through its capacity to create a partially solid gel matrix that envelops the starch, so reducing its digestion. It diminishes the amount of moisture available for starch gelatinization. Defatted rice bran is a substantial source of dietary fiber that forms a protective layer around starch, shielding it from amylolytic enzymes. As a result, the release of free glucose is hindered, leading to a diminished glycemic response.	[118]
	Gluten-free noodles was made of pregelatinized rice flour incorporated with germinated chickpea flours (5–30%).	The gluten-free noodles showed a significant reduction in glycemic index from 70.83 to 61.79 with better cooking quality.	The decrease in the GI of enriched noodles can potentially be attributed to several factors. There may be an increase in the concentration of RS, protein, and fiber in these noodles. The digestibility of noodle starch may be reduced by the development of complexes between protein and starch, as well as the formation of cross-links involving short chains of indigestible starch during the manufacture of the noodles.	[119]
	The effects of replacing a portion of rice flour (0–60%) with buckwheat flour (BF) on the in vitro starch digestibility of extruded rice-buckwheat noodles were examined.	Extruded noodles with 60% BF had an eGI of 67, which was still remarkably high and could not be desirable for nutritional items like rice-buckwheat noodles, even though the eGI fell from 85 to 67 with increasing proportions of BF.	For human consumption, the noodles utilized in the digestion experiment were subjected to both extrusion cooking and boiling water cooking techniques. The application of these two heating and cooling cycles led to an elevated eGI in the noodles. The enhancement of gel network formation during the process of extrusion cooking and its subsequent retrogradation have a notable impact on the digestion of starch. Noodles containing 30 g/100 g BF exhibited the formation of an integrated and entangled starch network. This network effectively hindered enzyme hydrolysis, leading to a reduced rate of digestion and absorption. Consequently, the noodles exhibited a considerably lower eGI.	[120]
	Matcha powder was fortified into noodle processing.	The addition of matcha significantly decreased RDS and eGI, as well as increased RS.	The interactions between polyphenols, starch, and proteins in MT could potentially disrupt the reassociation of starch chains, resulting in the creation of low-ordered crystalline structures. Additionally, scanning electron microscopy has provided evidence of the formation of dense microstructures. Consequently, rice noodles exhibited reduced cooking losses, increased chewability, and enhanced stretchability.	[63]
	Rice flour was replaced by finger millet (FM, 0–30%) in processing of composite rice noodles.	The RS content was 16.22 times greater in the sample with 30% substitution of finger millet than that of the control sample.	The presence of a significant amount of starch, protein, and dietary fiber in FM resulted in the formation of intermolecular connections that acted as a barrier, impeding the accessibility of α-amylase.	[121]
	Ultrasound-assisted cellulase enzymatic rice flour was used for preparation of rice noodles.	The GI value of the treated rice noodles (71.86) was significantly lower than that of the control (78.18).	This reduction may be attributed to the presence of proteins, lipids, and dietary fibers, which might potentially encapsulate starch granules or induce structural modifications in the starch. Consequently, these alterations may render the starch less susceptible to digestion.	[19]
	Common vetch (*Vicia sativa* L.) starch (CVS) was substituted for rice noodle formulation.	20% CVS substitution could improve the best texture quality of the rice noodles and reduce the eGI value (from 87.43 to 84.58).	This phenomenon can be attributed to the development of more relaxed gel structures and less organized structures, as the RS granules were shown to be more readily disassembled into distinct components by a significant quantity of CVS with bigger granule sizes. Additionally, RS containing a higher proportion of short chains exhibited a greater tendency to undergo cross-linking with other RS molecules during the process of retrogradation. As the level of CVS substitution increased, there was a corresponding decrease in the eGI of rice noodles, followed by a stabilization of the eGI.	[122]
	The complexation between rice noodles with xanthan gum and dodecyl gallate (DG) was investigated.	The synergistic effects of xanthan gum and DG could increase in SDS and RS and reduce eGI values.	The observed phenomena align with the patterns of alterations in crystalline structure, suggesting that DG molecules not only interacted with starch to facilitate the creation of V-type inclusion complexes, but also facilitated the reassembly of starch molecules during retrogradation. The increase in ordered aggregation structures of noodles can be attributed to the creation of starch DG inclusion and the reassociation of starch molecules. This phenomenon leads to the production of a smaller mesh size.	[93]
	Lauric acid (LA), myristic acid (MA), palmitic acid (PA), stearic acid (SA), oleic acid (OA), and linoleic acid (LOA) were incorporated into instant rice noodles (IRN).	Following the addition of LA, MA, PA, SA, OA, and LOA, respectively, the RS of IRN was noticeably reduced from the original value of 96.52% to 90.72%, 91.73%, 89.04%, 89.93%, 89.78%, and 88.55%.	The presence of double bonds in unsaturated fatty acids causes the carbon chains to become bent. The number of carbons available for complex formation in unsaturated fatty acids is decreased compared to saturated fatty acids. This drop is more pronounced in unsaturated fatty acids that include two double bonds. Therefore, it is likely that the curved carbon chains of unsaturated fatty acids enhanced their interaction with starch chains, despite the presence of greater steric hindrance compared to saturated fatty acids. The enhanced hydrophilicity exhibited by double bonds in comparison to saturated bonds, along with their increased solubility in water, facilitates the formation of lipid complexes in a more efficient manner.	[62]

## 5. Conclusions and Prospects

The report of the International Diabetes Federation stated the following: “537 million adults (20–79 years) are living with diabetes—1 in 10. This number is predicted to rise to 643 million by 2030 and 783 million by 2045” [1]. Furthermore, it is reported that diabetes is responsible for 6.7 million deaths in 2021. Additionally, there has been an increase in the prevalence of other digestive-related illnesses, like hypertension, obesity, atherosclerosis, colon cancer, and cardiovascular conditions, in recent years. It is thought that the prevalence of high or unstable blood sugar levels over protracted periods of time contributes to the pathophysiology of many chronic diseases. A vital component of the human diet is made up of the starchy foods that give the body glucose. To improve public health, novel starchy foods with a lower GI should be meticulously designed and prepared. Additionally, millions of people all around the world include rice in their everyday diets in enormous amounts. The challenge is to provide rice products in the best way feasible to promote health benefits due to the different effects of internal and external factors on the GI of rice and its products. Brown rice and colored rice could potentially have a low or medium–low GI; however, white rice has the higher digestion behavior due to many mechanisms. Understanding the mechanism of rice starch digestion in various conditions and the food matrix is critical to evaluate and formulate the nutritional and nutraceutical ingredients/products. The results from the review suggest that brown rice or colored rice could potentially be used for diabetic patients, and the composition of product and processing influenced digestion behavior. However, it also mentioned various advanced technologies could modulate the GI of the product. Therefore, based on this basic knowledge, the suggestion of lowering GI methods could be combined and optimized. Furthermore, information about *in vivo* tests also needs to be provided to better understand the effect on human metabolism. Additionally, the addition of different ingredients to the formula for rice products could lead to a variation in the digestion rate. To establish a larger study database for upcoming efforts on decreasing glycemic effects, it is possible to conduct scientific studies on these components and foods. Additionally, the technologies used need to be optimized to ensure economy while still creating quality products, which is the main consideration of food scientists and the industry.

## Data Availability

No data available in this article.
